# Efficacy of an emotion and stress management program based on empowerment theory on family caregivers of stroke survivors

**DOI:** 10.12669/pjms.41.2.11350

**Published:** 2025-02

**Authors:** Tengteng Liu, Runhong Zhang, Hongying Yao, Lan Que, Weiwei Bai

**Affiliations:** 1 Tengteng Liu Department of Otolaryngology, Linping Campus, The Second Affiliated Hospital of Zhejiang University School of Medicine, Hangzhou, Zhejiang Province 311100, P.R. China; 2 Runhong Zhang Department of Otolaryngology, Linping Campus, The Second Affiliated Hospital of Zhejiang University School of Medicine, Hangzhou, Zhejiang Province 311100, P.R. China; 3 Hongying Yao Department of Nursing, Linping Campus, The Second Affiliated Hospital of Zhejiang University School of Medicine, Hangzhou, Zhejiang Province 311100, P.R. China; 4 Lan Que Department of Nursing, Linping Campus, The Second Affiliated Hospital of Zhejiang University School of Medicine, Hangzhou, Zhejiang Province 311100, P.R. China; 5 Weiwei Bai Department of Neurosurgery, Linping Campus, The Second Affiliated Hospital of Zhejiang University School of Medicine, Hangzhou, Zhejiang Province 311100, P.R. China

**Keywords:** Emotional and stress management, Empowerment theory, Family caregivers, Stroke survivors

## Abstract

**Objective:**

This study aimed to analyze the efficacy of an emotion and stress management program based on empowerment theory on family caregivers of stroke survivors.

**Methods:**

This retrospective study was conducted at Linping Campus, The Second Affiliated Hospital, Zhejiang University School of Medicine, and included 130 family caregivers of stroke survivors from October 2022 to May 2024. The caregivers were given emotion and stress management program based on empowerment theory (Empowerment group, n=65) or given traditional management program (Traditional group, n=65). Caregiver burden inventory (CBI), self-rating depression scale (SDS), self-rating anxiety scale (SAS), and the Chinese version of the Perceived Stress Scale (CPSS) were used for analysis.

**Results:**

After intervention, CBI, SDS, SAS, and CPSS in both groups decreased significantly compared to before intervention and were significantly lower in the Empowerment group compared to the Traditional group (*P*<0.05).

**Conclusions:**

Compared with traditional management, emotional and stress management plans based on empowerment theory can reduce caregiving burden, alleviate negative emotions, and improve perceived stress in family caregivers of stroke survivors.

## INTRODUCTION

Stroke is considered the second leading cause of mortality and is associated with considerable rates of long-term disability.^1^ Approximate 75% of stroke survivors may have varying degrees of sequelae,^2^,^3^ and the disability rate among stroke survivors can reach 38.2% to 62.8% one year after the onset.^4^ Stroke survivors require long-term rehabilitation training after discharge from hospital, which imposes a heavy burden on their families, leading to depression and anxiety in caregivers.^5^ The caregivers’ physical and mental state can have a certain impact on the recovery and quality of life of stroke survivors.^5^,^6^ While there have been many studies on the rehabilitation management of stroke survivors in clinical settings, research on the physical and mental state of family caregivers is scarce.^3^,^4^

Empowerment theory refers to intervention methods that integrate social policies and support systems to stimulate the subjective initiative of individuals, encouraging them to face negative factors actively and regulating their physical and mental state.^7^,^8^ Empowerment theory has been shown to play an important role in managing various chronic diseases, strengthening the individual resilience of patients, and guiding them to regulate negative emotions.^8^,^9^

However, the effectiveness of emotion and stress management program based on empowerment theory in family caregivers of stroke survivors has not been widely confirmed in current clinical practice. This study aimed to compare the effects of traditional management program with emotion and stress management program based on empowerment theory in family caregivers of stroke survivors.

## METHODS

**Fig.1:**
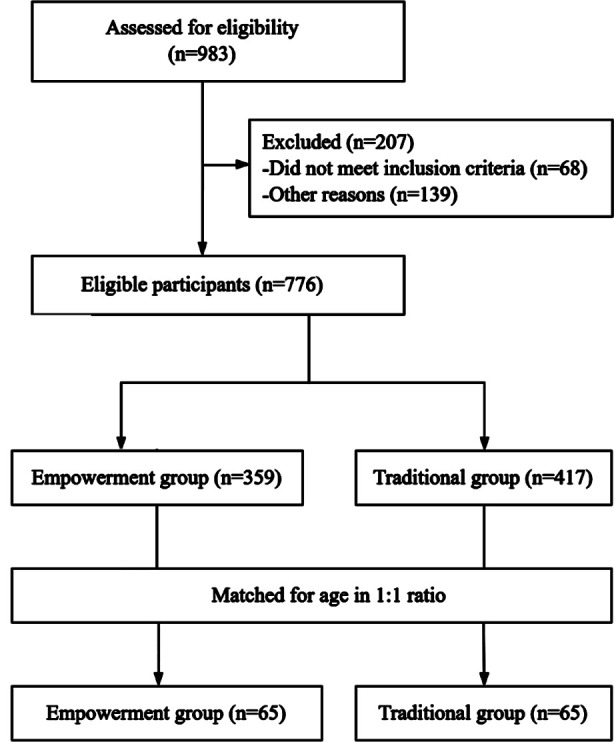
Participants inclusion and exclusion criteria flowchart; n = pairs of patients and their family caregivers.

This retrospective study was conducted at the Linping Campus, The Second Affiliated Hospital, Zhejiang University School of Medicine, and included 130 family caregivers of stroke survivors from October 2022 to May 2024. Among them, 65 people received an emotional and stress management program based on empowerment theory (Empowerment group) and 65 patients received a traditional management plan (Traditional group). The flowchart of participants were shown in Fig.1.

### Ethical Approval:

This study was approved by the Institutional Review Board of Linping Campus, The Second Affiliated Hospital, Zhejiang University School of Medicine, (IRB approval number 2022-084, Date: December 9, 2022).

### Inclusion criteria:

For patients, the inclusion criteria were as follows:

- Patients with onset of first stroke confirmed by CT or MRI;
- Age≥40 years old;
- Patient with stable condition and had no disturbance of consciousness;
- Patients with family caregivers;
- Patients with Barthel Index for Activities of Daily Living between 20-60.

### Exclusion criteria:

- Patients with other severe diseases such as malignant tumors, heart failure and renal failure.
- Individuals with mental disorders or cognitive impairment.

### For caregivers, the inclusion criteria were as follows:

- Age≥18 years old.
- Patient’s children, spouse, or parents
- Care duration ≥ 4 h/d and ≥ 12 weeks
- Had good reading comprehension and communication skills.

### The exclusion criteria were as follows:

- Professional caregivers.
- Individuals with mental disorders.
- Individuals with disabilities or benign or malignant tumors.

### Traditional group:

The traditional management program was summarized in Table-I.

**Table-I:** Traditional Management Program.

Time	Content	Implementation form	Implementation timing
On admission	1) Introduction to the hospital environment and medical staff;	Oral	On the day of admission
	2) Detailed explanation of the causes, clinical manifestations, pathogenesis, daily nursing measures, and precautions of stroke;		
	3) Clarifying the needs of patients and family caregivers and providing timely assistance.		
During hospitalization	1) Providing detailed information on the patient’s condition;	Oral	During hospitalization
	2) Patiently listening to the description of the patient’s illness by the family caregiver;		
	3) Guiding family caregivers to master the clinical manifestations and emergency measures of stroke onset;		
	4) Explaining the usage and dosage of medication, as well as the necessity of strictly following medical advice;		
At discharge	1) Explaining to family caregivers the names, usage, and dosage of medications required for discharge;	Oral	At discharge
	2) Reminding family caregivers to take patients for follow-up examinations regularly;		
	3) Reminding family caregivers to supervise patients’ regular sleep and diet;		
	4) Informing family caregivers to care for and accompany patients outside the hospital and enhance communication;		
Follow-up	1) Mastering the patient’s home rehabilitation situation;	Follow-up	2^nd^ weeks, 8^th^ weeks, and 12^th^ weeks after discharge
	2) Identifying the problems that exist during home rehabilitation and providing timely assistance and guidance.		

### Empowerment group:

A team consisting of a neurologist, rehabilitation physician, head nurse, and head nurse was established to develop, evaluate and implement the emotion and stress management program based on the empowerment theory. Specific measures of the emotion and stress management program based on the empowerment theory were summarized in Table-II.

**Table-II:** The Emotion and Stress Management Program Based on the Empowerment Theory.

Week/Location	Intervention theme	Empowering intervention nodes	Concrete measure	Family tasks
First week (face-to-face)	Get to know each other	Identifying problems, and build good relationships between caregivers and family caregivers	1) Self-introduction; 2) Master the caregiver’s family situation through communication and exchange; 3) Elaborate on the value of this intervention plan for patients and caregivers; 4) Master the problems and needs faced by caregivers;	No
Second week (face-to-face)	Emotional expression	Expressing emotions and patiently listening to caregivers’ emotions and attitudes toward the patient’s illness	1) Encourage participants to express their subjective feelings actively; 2) Provide unconditional support, respect, and empathy to caregivers 3) Guide caregivers to express their emotions towards patients and their families;	Take appropriate measures to express emotions to patients and their families
Third week (face-to-face)	Inspire intrinsic motivation	Expressing emotions and actively expressing the intrinsic motivation to take care of patients	1) Explain to caregivers the concept of intrinsic motivation and the types of factors that stimulate intrinsic motivation; 2) Encourage caregivers to express their intrinsic motivation to care for patients; 3) Guide caregivers to explore motivational factors and strengthen self-efficacy;	Reflect on and express the intrinsic motivation for caring for patients
Fourth week (face-to-face/online)	Have a clear goal and program	Set goals, clarify goals and plans for dealing with difficulties, strengthen intrinsic motivation, establish plans and specific plans	1) Agree with the caregiver system on goals to enhance intrinsic motivation, regulate emotions, and reduce stress; 2) Discuss solutions to regulate stress; 3) Encourage caregivers to express ways to relieve emotions; 4) Clarify the ultimate goal and achieve mastery of ways to relieve stress and regulate emotions;	Understand and reflect on goals, enhance health beliefs
Fifth week (face-to-face/online) e)	Emotional Coping	Implement the plan and effectively deal with emotions	1. Explain the negative impact of negative emotions on physical and mental health; 2. Encourage caregivers to express their venting measures when feeling down; 3. Assist caregivers in developing effective ways of emotional release;	Choose appropriate ways to vent negative emotions when they arise emotions when they arise
Sixth week (Face to face/online)	Emotional acceptance	Implement the plan and accept emotions	1) Identify negative emotions; 2) Guide caregivers to have a correct understanding of their own emotional coping measures; 3) Guide and assist caregivers in engaging in emotional dialogue;	Correctly adopting emotional externalization techniques and emotions
Eighth week (Online)	Cognitive on stress	Implement the plan and learn to regulate cognition	1) The correlation between educational emotions and stress; 2) Explore the essence of stress in caring for patients; 3) Inform caregivers that cognition is an important mediator of stress formation; 4) Guide caregivers to change their cognition and engage in reasonable emotional imagination exercises;	Carry out reasonable emotional imagination exercises
Tenth week (online)	Relax both body and mind	Implement the plan and master relaxation techniques	1) Carry out progressive muscle relaxation training; 2) Demonstrate common relaxation methods in daily life and guide caregivers to choose suitable relaxation activities for themselves; 3) Guide caregivers to learn body relaxation techniques such as self-massage of the head, feet, or hands;	Perform 20-30 minutes of relaxation exercises daily
Twelfth week (online)	Consolidate Practice	Evaluate the results, express feelings, and assess whether the intervention goals have been achieved	1) The caregiver expresses the muscle progressive approach; 2) Changes in emotions and stress expressed by caregivers; 3) Guide caregivers to summarize effective measures to relieve stress and regulate emotions.	Reasonably adopt emotional and stress regulation measures

### Observation indicators:

- Care burden. It was assessed by caregiver burden inventory (CBI), which includes five dimensions of caregiver burden: time-dependence (20 points), developmental (20 points), physical (16 points), social care (16 points), and emotional dimensions (24 points). There are a total of 24 items, each worth 0-4 points, and the higher the score, the heavier the burden on caregivers.^10^
- Negative emotion. Depression and anxiety were assessed by the self-rating depression scale (SDS) and self-rating anxiety scale (SAS), respectively. Both the SDS and SAS scales consist of 20 items, each rated on a scale of 1-4 based on feelings from the past week. After adding the scores of each item to obtain a rough score, the rough score is multiplied by 1.25 to obtain the standard score. A higher score indicates a higher level of depression or anxiety.^11^
- Stress. It was assessed by the Chinese version of the Perceived Stress Scale (CPSS).The CPSS consists of 14 items reflecting feelings of stress, tension, and loss of control.^12^ The scale uses a five point scoring system, with a higher total score indicating greater pressure.

### Statistical Analysis:

All analyses were conducted using SPSS 25.0 software (IBM, Armonk, New York, USA). Continuous variables were reported as mean and standard deviation (SD) or median and interquartile range (IQR), based on the normality of distribution, assessed by the Shapiro-Wilk test. The statistical significance of differences in continuous variables between the Empowerment group and Traditional group was assessed using student’s t-tests for normally distributed data and Mann-Whitney U tests for non-normally distributed data. Paired t-test was used for normal distribution data and Wilcoxon signed rank test was used for non-normal distribution data to evaluate the statistical significance of the differences in continuous variables before and after intervention in the Empowerment group and Traditional group. For categorical variables, frequency distribution with percentage was provided, and Chi-square test was used to compare categorical variables between two groups. *P*<0.05 was considered statistically significant.

## RESULTS

A total of 130 family caregivers were included and each group had 65 caregivers. There were no significant differences in the baseline data, such as age, gender, marital status, patient relationship, education level, average monthly household income, and place of residence between the two groups of caregivers (*P*>0.05) (Table-III).

**Table-III:** Comparison of demographics of caregivers and patients between the two groups.

Baseline data	Empowerment group (n=65)	Traditional group (n=65)	t/χ^2^	P
***Caregivers***				
Age (years), mean±SD	57.06±7.56	55.66±6.40	1.140	0.256
Male (yes), n(%)	23 (35.4)	26 (40.0)	0.295	0.587
***Marital status, n (%)***				
Married	51 (78.5)	54 (83.1)	0.446	0.504
Unmarried/divorced/widowed	14 (21.54)	11 (16.92)		
***Relationship with patients, n (%)***				
Spouse	24 (36.9)	21 (32.3)	0.788	0.674
Children	25 (38.5)	30 (46.2)		
Parent	16 (24.6)	14 (21.5)		
***Educational level, n (%)***				
Junior high school and below	48 (73.8)	55 (84.6)	2.291	0.130
High school and above	17 (26.2)	10 (15.4)		
***Per capita monthly household income (yuan), n (%)***				
<5000	24 (36.92)	26 (40.00)	0.155	0.926
≥5000	9 (13.85)	8 (12.31)		
Place of residence, n (%)				
Town	25 (38.5)	20 (30.8)	0.850	0.357
Countryside	40 (61.5)	45 (69.2)
***Patients***				
Age (years), mean±SD	67.51±6.39	65.58±7.58	1.564	0.120
Barthel Index for Activities of Daily Living (score), mean±SD	41.75±7.37	43.31±6.18	-1.303	0.195

SD: standard deviation.

Before intervention, the scores of time dependence care burden, developmental care burden, physical care burden, social care burden, and emotional care burden were similar in the two groups (*P*>0.05). After the intervention, both groups showed a significant decrease in scores, and the scores of the Empowerment group were significantly lower than those of the Traditional group (P<0.05) (Table-IV).

**Table-IV:** Comparison of CBI scores between two groups, M(P25/P75)

Variable	Empowerment group (n=65)	Traditional group (n=65)	Z	P
***Before treatment***				
Time dependence care burden	15(14-17)	15(14-16)	-1.458	0.145
Developmental care burden	9(8-11)	9(8-10)	-1.279	0.201
Physical care burden	10(9-11)	10(9-11)	-1.243	0.214
Social care burden	11(10-12)	12(10-13)	-0.659	0.510
Emotional care burden	12(11-14)	12(10-13)	-1.182	0.237
***After treatment***				
Time dependence care burden	8(8-9)^a^	9(8-12)^a^	-3.425	0.001
Developmental care burden	6(6-8)^a^	8(8-9)^a^	-5.051	<0.001
Physical care burden	6(5-7)^a^	8(7-9)^a^	-5.671	<0.001
Social care burden	7(6-8)^a^	9(7-10)^a^	-4.254	<0.001
Emotional care burden	8(6-9)^a^	9(8-11)^a^	-6.195	<0.001

***Note:*** Compared with before treatment in the same group,

a P<0.05; CBI: caregiver burden inventory; SD: standard deviation.

Before intervention, there was no significant difference in SDS, SAS, and CPSS scores between the two groups (*P*>0.05). After intervention, the scores of both groups decreased significantly compared to pre-intervention values, and were significantly lower in the Empowerment group compared to the Traditional group (*P*<0.05) (Table-V).

**Table-V:** Comparison of SDS, SAS, and CPSS scores between two groups;

Variable	Empowerment group (n=65)	Traditional group (n=65)	t/Z	P
***Before treatment***				
SDS, mean±SD	58.43±4.91	59.69±4.52	-1.523	0.130
SAS, M(P25/P75)	62(58-64)	60(54-64)	-1.566	0.117
CPSS, M(P25/P75)	39(36-44)	41(38-45)	-1.264	0.206
***After treatment***				
SDS, mean±SD	40.29±3.55^a^	46.03±4.91^a^	-7.633	<0.001
SAS, M(P25/P75)	41(38-43)^a^	46(42-51)^a^	-4.964	<0.001
CPSS, M(P25/P75)	32(30-36)^a^	39(35-43)^a^	-5.546	<0.001

***Note:*** Compared with before treatment in the same group,

a P<0.05; SDS: self-rating depression scale; SAS: self-rating anxiety scale; CPSS: Chinese version of the perceived stress scale; SD: standard deviation.

## DISCUSSION

This study showed that compared with the traditional management approach, a program that based on the empowerment theory was associated with a lighter caregiving burden, lower levels of depression and anxiety, and less stress among the caregivers of stroke survivors. The care of stroke survivors is a continuous and complex long-term process. After the discharge of the stroke survivors, family caregivers are responsible for bearing all associated burdens. Caregivers may experience a decline in their quality of life due to changes in their roles, emotional stress, and work pressure. Meanwhile, the physical and mental health status of family caregivers can directly affect the rehabilitation outcomes and quality of life of stroke patients.^13^,^14^ Positive nursing experience can make family caregivers more confident and somewhat reduce their caregiving burden.^15^ Lobo et al.^16^ showed that the previous approaches mainly used hands-on teaching and only provided oral education to family caregivers, often with unsatisfactory effects. This study implemented an emotional and stress management program based on the empowerment theory to intervene with family caregivers of stroke survivors and showed that the Empowerment group had lower scores in all dimensions of care burden, SDS, SAS, and CPSS, compared to the Traditional group. Our results further confirmed the clinical value of emotion and stress management programs based on the empowerment theory and its efficacy in regulating the physical and mental state of family caregivers of stroke survivors, which is consistent with previous studies.^17^,^18^

The empowerment theory divides the intervention process into five stages: problem identification, emotional expression, goal setting, plan formulation, and outcome evaluation. During the intervention period, both parties have an equal relationship, which can effectively stimulate individual subjective initiative and encourage the intervention subjects to develop good behavior.^19^,^20^ Tao et al.^21^ showed that health education based on empowerment theory for elderly patients with diabetes could improve patients’ treatment compliance, self-management ability, blood sugar level, and quality of life. Lee et al.^22^ also indicated that providing emotional support or stress relief strategy counseling to family caregivers of stroke survivors can alleviate their caregiving burden. Kusmaul et al.^23^ demonstrated that during the intervention period of empowerment theory, emphasis is placed on emotional release, and communication and interaction are enhanced, as they are based on building a good relationship with the intervention subjects. Such an approach helps them to recognize and accept negative emotions, explore breakthrough points for emotional expression, release tension, and improve their cognitive and regulation levels. It is plausible that emotional and stress management programs based on empowerment theory can effectively improve the psychological state of family caregivers and reduce psychological stress, thus reducing the negative impact of care for stroke patients on the physical and mental health of family members-caregivers.^24^,^25^

### Limitations:

Firstly, this is a single-center retrospective analysis with a small sample size, which may result in some bias. Secondly, while the learning ability of each family caregiver is different, the effect of transferring intervention measures to patients has not been analyzed. Thirdly, hospital wards (routine care or intensive care units) and the length of hospital stay may also affect the mental health of the caregivers. Further research is needed to develop specific strategies suitable for Chinese family caregivers to alleviate their burden of caring for stroke survivors.

## CONCLUSION

An emotional and stress management program based on empowerment theory for family caregivers of stroke survivors can reduce their caregiving burden, alleviate negative emotions, improve perceived stress levels, and achieve high satisfaction. Implementing such a program may potentially contribute not only to improving the well-being of the caregivers but also to better functional outcomes and quality of life of stroke survivors.

### Author’s contributions:

**TL:** Study concept, design, Literature search, and manuscript writing.

**RZ**, **HY**, **LQ** and **WB:** Data collection, data analysis, interpretation and critical review.

**TL:** Manuscript revision and validation and is responsible for the integrity of the study.

All authors have read and approved the final manuscript.

## References

[1] Katan M, Luft A (2018). Global Burden of Stroke. Semin Neurol.

[2] Saini V, Guada L, Yavagal DR (2021). Global Epidemiology of Stroke and Access to Acute Ischemic Stroke Interventions. Neurology.

[3] Liao Z, Liao F (2024). Effect of early pulmonary rehabilitation therapy on the pulmonary function of patients with stroke-associated pneumonia and analysis of its effectiveness. Pak J Med Sci.

[4] Wafa HA, Wolfe CDA, Emmett E, Roth GA, Johnson CO, Wang Y (2020). Burden of Stroke in Europe:Thirty-Year Projections of Incidence, Prevalence, Deaths, and Disability-Adjusted Life Years. Stroke.

[5] Kwakkel G, Stinear C, Essers B, Munoz-Novoa M, Branscheidt M, Cabanas-Valdés R (2023). Motor rehabilitation after stroke:European Stroke Organisation (ESO) consensus-based definition and guiding framework. Eur Stroke J.

[6] Molu NG, Ozkan B, Icel S (2016). Quality of life for chronic psychiatric illnesses and home care. Pak J Med Sci.

[7] Thitipitchayanant K, Somrongthong R, Kumar R, Kanchanakharn N (2018). Effectiveness of self-empowerment-affirmation-relaxation (Self-EAR) program for postpartum blues mothers:A randomize controlled trial. Pak J Med Sci.

[8] Morris L, Mansell W, Williamson T, Wray A, McEvoy P (2020). Communication Empowerment Framework:An integrative framework to support effective communication and interaction between carers and people living with dementia. Dementia (London).

[9] Saban KL, Tell D, De La Pena P (2022). Nursing Implications of Mindfulness-Informed Interventions for Stroke Survivors and Their Families. Stroke.

[10] Strini V, Prendin A, Cerrone V, Schiavolin R, De Barbieri I, Andretta V (2023). Scale of Assessment of Caregiver Care Burden of People with Dementia:A Systematic Review of Literature. Transl Med UniSa.

[11] Zhao X, Sha X, Qi L (2024). Effects of comprehensive exercise training on frailty, negative emotions and physical functions of elderly patients with diabetes. Pak J Med Sci.

[12] She Z, Li D, Zhang W, Zhou N, Xi J, Ju K (2021). Three Versions of the Perceived Stress Scale:Psychometric Evaluation in a Nationally Representative Sample of Chinese Adults during the COVID-19 Pandemic. Int J Environ Res Public Health.

[13] Pont W, Groeneveld I, Arwert H, Meesters J, Mishre RR, Vliet Vlieland T (2020). Caregiver burden after stroke:changes over time?. Disabil Rehabil.

[14] Caunca MR, Simonetto M, Hartley G, Wright CB, Czaja SJ (2020). Design and Usability Testing of the Stroke Caregiver Support System:A Mobile-Friendly Website to Reduce Stroke Caregiver Burden. Rehabil Nurs.

[15] Tyagi S, Koh GCH, Luo N, Tan KB, Hoenig H, Matchar DB (2020). Dyadic approach to supervised community rehabilitation participation in an Asian setting post-stroke:exploring the role of caregiver and patient characteristics in a prospective cohort study. BMJ Open.

[16] Lobo EH, Frølich A, Kensing F, Rasmussen LJ, Livingston PM, Grundy J (2021). mHealth applications to support caregiver needs and engagement during stroke recovery:A content review. Res Nurs Health.

[17] Deyhoul N, Vasli P, Rohani C, Shakeri N, Hosseini M (2020). The effect of family-centered empowerment program on the family caregiver burden and the activities of daily living of Iranian patients with stroke:a randomized controlled trial study. Aging Clin Exp Res.

[18] Dharma K K, Damhudi D, Yardes N, Haeriyanto S (2021). Caregiver empowerment program based on the adaptation model increase stroke family caregiver outcome. Front Nursing.

[19] Cuzco C, Delgado-Hito P, Marin-Pérez R, Núñez-Delgado A, Romero-García M, Martínez-Momblan MA (2023). Transitions and empowerment theory:A framework for nursing interventions during intensive care unit patient transition. Enferm Intensiva (Engl Ed).

[20] Vainauskienė V, Vaitkienė R (2021). Enablers of Patient Knowledge Empowerment for Self-Management of Chronic Disease:An Integrative Review. Int J Environ Res Public Health.

[21] Tao Y, Wang Y (2023). Effect of empowerment theory health education on disease control level and compliance of elderly T2DM. Pak J Pharm Sci.

[22] Lee JW, Sohn MK, Lee J, Kim DY, Shin YI, Oh GJ (2024). Predictors of Burden for First-Ever Stroke Survivor's Long-Term Caregivers:A Study of KOSCO. Medicina (Kaunas).

[23] Kusmaul N, Butler S, Hageman S (2020). The Role of Empowerment in Home Care Work. J Gerontol Soc Work.

[24] Raemdonck E, Lambotte D, De Witte N, Gorus E (2022). Giving voice to informal caregivers of community-dwelling older adults:A systematic review of empowerment interventions. Health Soc Care Community.

[25] McCreary DDJ (2020). Home Health Nursing Job Satisfaction and Retention:Meeting the Growing Need for Home Health Nurses. Nurs Clin North Am.

